# Loop-Mediated Isothermal Amplification for *Salmonella* Detection in Food and Feed: Current Applications and Future Directions

**DOI:** 10.1089/fpd.2018.2445

**Published:** 2018-06-01

**Authors:** Qianru Yang, Kelly J. Domesle, Beilei Ge

**Affiliations:** Division of Animal and Food Microbiology, Office of Research, Center for Veterinary Medicine, U.S. Food and Drug Administration, Laurel, Maryland.

**Keywords:** LAMP, *Salmonella*, detection, food, feed

## Abstract

Loop-mediated isothermal amplification (LAMP) has become a powerful alternative to polymerase chain reaction (PCR) for pathogen detection in clinical specimens and food matrices. Nontyphoidal *Salmonella* is a zoonotic pathogen of significant food and feed safety concern worldwide. The first study employing LAMP for the rapid detection of *Salmonella* was reported in 2005, 5 years after the invention of the LAMP technology in Japan. This review provides an overview of international efforts in the past decade on the development and application of *Salmonella* LAMP assays in a wide array of food and feed matrices. Recent progress in assay design, platform development, commercial application, and method validation is reviewed. Future perspectives toward more practical and wider applications of *Salmonella* LAMP assays in food and feed testing are discussed.

## Introduction

Nontyphoidal *Salmonella* is a Gram-negative zoonotic pathogen of substantial public health concern (WHO, 2017). In the 2015 World Health Organization (WHO) estimates of the global burden of foodborne diseases, *Salmonella* ranked first among 22 bacterial, protozoal, and viral agents, reflecting its ubiquitous nature and the severity of illnesses (Kirk *et al.*, 2015).

In the United States, over 75% of *Salmonella* outbreak-associated illnesses were broadly attributed across multiple food categories, including produce, eggs, chicken, pork, and beef (IFSAC 2015, 2017). *Salmonella* is also recognized as a major microbial hazard in animal food, which includes pet food, animal feed, and raw materials and ingredients (EFSA, 2008; FAO/WHO, 2015; FDA, 2017b). Multistate outbreaks of human salmonellosis linked to tainted pet food have been reported (CDC, 2018). Moreover, some *Salmonella* serovars are also major animal pathogens, for example, *Salmonella* Dublin in cattle and *Salmonella* Gallinarum in poultry, resulting in considerable loss in livestock production (Uzzau *et al.*, 2000; FDA, 2013).

To prevent or reduce *Salmonella* outbreaks/illnesses from contaminated human or animal food, vigilant product testing and environmental monitoring for pathogens are critical, as underscored by the Food Safety Modernization Act (FSMA) regulations on preventive controls (FDA, 2017a, b). This highlights the importance and urgency to develop rapid, reliable, and robust methods for *Salmonella* detection in a variety of food and feed matrices.

According to a recent report, the global food microbiology testing for pathogens totaled 280 million tests in 2016, a market valued at $1.8 billion (Ferguson, 2017). This represents an increase of 23.2% in testing volume over a 3-year period. Not surprisingly, *Salmonella* was the target in 43% of all tests performed, followed by *Listeria* and *Listeria monocytogenes* (41%), pathogenic *Escherichia coli* (14%), and *Campylobacter* (2%). A clear shift from traditional methods to rapid methods (e.g., polymerase chain reaction [PCR]) has been the trend observed for all four priority pathogens in the past two decades (Ferguson, 2017).

Loop-mediated isothermal amplification (LAMP) (Notomi *et al.*, 2000) is a novel nucleic acid amplification test (NAAT) that has recently emerged as a powerful alternative to PCR for the rapid detection of various bacterial, fungal, parasitic, and viral agents (Niessen *et al.*, 2013; Li *et al.*, 2017). The first LAMP assay targeting *Salmonella* was reported in 2005 (Hara-Kudo *et al.*, 2005). Since then, dozens of new *Salmonella* LAMP assays have been developed, leading to broad applications in human food and more recently in animal feed.

This review aims to capture international efforts in the past decade on the development and application of *Salmonella* LAMP assays in food and feed matrices. Future perspectives toward even more practical and wider applications of such assays in food and feed testing are discussed.

## LAMP in a Nutshell

LAMP was invented in 2000 by a group of Japanese scientists (Notomi *et al.*, 2000). The mechanism is based on the production of a stem-loop DNA structure during initiation steps, which serves as the starting material for second-stage LAMP cycling (refer to this site (Eiken Chemical Co. Ltd., 2005) for LAMP diagrams and animation). Unlike PCR (Table 1) that relies on thermal cycling to denature DNA and enable amplification by *Taq* DNA polymerase, LAMP uses a strand-displacing *Bst* DNA polymerase, which allows autocycling amplification under a constant temperature (60–65°C). This obviates the need for a sophisticated thermocycler. There are four to six specially designed LAMP primers (Nagamine *et al.*, 2002), which target six to eight regions of the template DNA, compared to two primers in PCR (plus one or more probes in real-time PCR where amplification and detection occur simultaneously), ensuring a highly specific assay.

**Table 1.** Technical Comparison Between Loop-Mediated Isothermal Amplification and Polymerase Chain Reaction (or Real-Time Polymerase Chain Reaction)

*Assay step*	*Component*	*LAMP*	*PCR or real-time PCR*
Amplification	Enzyme	*Bst* DNA polymerase or equivalent ones	*Taq* DNA polymerase or equivalent ones
		High strand displacement activity	Thermal cycling requirement (95°C/55°C/72°C)
		Autocycling DNA amplification	
		Isothermal (60–65°C)	
	Primer	Four to six, two are longer ones (double length, ∼40 bp)	Two, plus one or more probes (real-time PCR)
	Other reagents	dNTP, buffer, Mg^2+^, water	dNTP, buffer, Mg^2+^, water
Detection	Platform	Gel electrophoresis, turbidity, naked eye, colorimetric, fluorescence, bioluminescence, etc.	Gel electrophoresis, fluorescence (real-time PCR)

LAMP, loop-mediated isothermal amplification; PCR, polymerase chain reaction.

LAMP amplifies the target DNA rather efficiently, with 10^9^ copies generated within an hour (Notomi *et al.*, 2000). PCR or real-time PCR generally takes 1–2 h (although speedier versions are available now) and the amount of DNA produced is almost 20 times less (Mashooq *et al.*, 2016). LAMP is highly tolerant to biological substances (Kaneko *et al.*, 2007) with robustness demonstrated in both clinical and food applications (Francois *et al.*, 2011; Yang *et al.*, 2014). PCR, on the other hand, is generally susceptible to various assay inhibitors present in complex food or feed matrices (Abu Al-Soud and Radstrom, 2000; Maciorowski *et al.*, 2005). LAMP is also more versatile in terms of amplicon detection methods, which include naked eye, colorimetry, turbidity, fluorescence, and bioluminescence, among many others (Zhang *et al.*, 2014).

These attractive features of LAMP appear to align well with the WHO-outlined ASSURED (which stands for affordable, sensitive, specific, user friendly, rapid and robust, equipment free, and delivered to those who need it) criteria for an ideal diagnostic test (Mabey et al., 2004). As such, LAMP has become a mainstream isothermal NAAT used for low-cost point-of-care (POC) diagnostics and has reached a high level of maturity (Niemz *et al.*, 2011; de Paz *et al.*, 2014). In August 2016, WHO issued a recommendation for a TB-LAMP (LAMP for detection of *Mycobacterium tuberculosis*) method as a rapid, accurate, and robust replacement test for smear microscopy to diagnose tuberculosis in peripheral health centers (WHO, 2016).

Applications of LAMP also extend to many other fields beyond *in vitro* diagnostics, as summarized in several recent reviews, such as species authentication and microbiological quality/safety assessment in meats (Kumar *et al.*, 2017), and testing for genetically modified organisms (GMOs), allergens, pesticides, and drug resistance (Kundapur and Nema, 2016). A quick PubMed search using the term “loop-mediated isothermal amplification” returned >2100 articles, highlighting the great interest in LAMP within the scientific community.

**FIG. 1.**
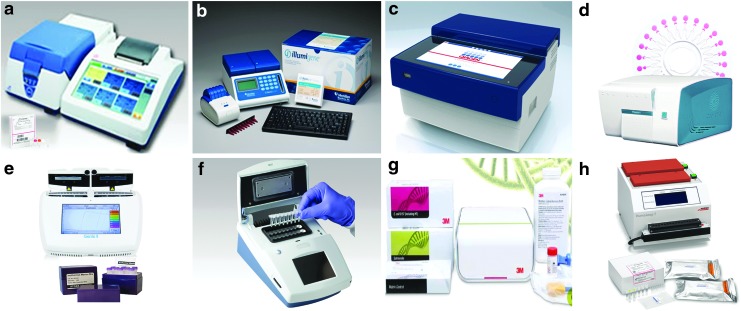
LAMP commercial applications. **(a)** Loopamp Realtime Turbidimeter LA-500 and reagent kits (Eiken Chemical Co., Ltd., Tokyo, Japan); **(b)** *illumipro-10* and *illumigene* Molecular Diagnostic System (Meridian Bioscience, Inc., Cincinnati, OH); **(c)** ESEQuant TS2 (Qiagen, Venlo, Netherlands); **(d)** RTisochip-A (CapitalBio Technology Co., Ltd., Beijing, China); **(e)** Genie II and reagents (OptiGene Ltd., West Sussex, United Kingdom); **(f)** PDQ (ERBA Molecular, Cambridgeshire, United Kingdom); **(g)** 3M Molecular Detection System and assays (3M Food Safety, St. Paul, MN); **(h)** HumaLoop T and assays (HUMAN Diagnostics, Wiesbaden, Germany). LAMP, loop-mediated isothermal amplification.

The popularity of LAMP is also reflected in the development of many commercially available systems (Fig. 1). Along with these exciting developments, the LAMP technology has been explored by researchers around the globe for the rapid, reliable, and robust detection of *Salmonella* in human food and animal food, which is the focus of this review.

## *Salmonella* LAMP Assay Development

Japanese scientists Hara-Kudo *et al.* (2005; Ohtsuka *et al.*, 2005) have pioneered the field of LAMP detection for *Salmonella* in terms of initial assay development and food applications. In 2005, they described the first *Salmonella* LAMP assay and its application in artificially inoculated as well as naturally contaminated liquid eggs (Hara-Kudo *et al.*, 2005; Ohtsuka *et al.*, 2005). Since 2008, dozens of new *Salmonella* LAMP assays (i.e., with newly designed primers) have been developed, many of which were summarized in two excellent reviews published in 2013 (Niessen *et al.*, 2013; Kokkinos *et al.*, 2014).

Table 2 presents our collection (through regular PubMed and Web of Science searches and active literature gathering for ongoing research) of all *Salmonella* LAMP studies (*n* = 100) reported to date, some focusing on new assay developments (46% of studies) or new platform developments (34%), and others on applications in food (63%) or feed matrices (6%). Notably, scientists in China (32% of studies), United States (29%), Korea (8%), and Japan (5%) have contributed most to the advancements in this field.

**Table 2.** A Chronological List of *Salmonella* Loop-Mediated Isothermal Amplification Assay Developments, Platform Developments, and Applications in Food and Feed

							*Sensitivity*		*Specificity*				*Sensitivity in matrix*			
*Study type*^a^	*Year*	*Country*^b^	*Target organism*	*Target gene*	*Platform*	*Detection*	*Pure culture*	*PCR comparison*^c^	*Inclusivity (No. of strains)*	*Exclusivity (No. of strains)*	*Matrix*	*Nature or spike*	*No enrichment*	*With enrichment*	*Agreement with culture or PCR*	*References*
1, 3	2005	Japan	*Salmonella* spp.	*invA*	Real-time thermal cycler (ABI7700)	Real-time fluorescence (YO-PRO-1 iodide); naked eye (turbidity); gel electrophoresis	2.2–18.5 CFU	10 ×	100% (227)	100% (62)	Liquid eggs	Spiked	2.8 CFU/test (560 CFU/mL)	N/A	N/A	Hara-Kudo *et al.* (2005)
3	2005	Japan	*Salmonella* spp.	*invA*	Real-time thermal cycler (ABI7700)	Real-time fluorescence (YO-PRO-1 iodide); naked eye (turbidity)	N/A	N/A	N/A	N/A	Liquid eggs	Natural	N/A	1–25 CFU/25 g	Superior than culture and PCR	Ohtsuka *et al.* (2005)
1	2008	China	*Salmonella* spp.	*invA*	Unspecified	Gel electrophoresis	100 fg	10 ×	100% (6)	100% (14)	N/A	N/A	N/A	N/A	N/A	Wang *et al.* (2008b)
1, 3	2008	China	*Salmonella* spp.	*invA*	Unspecified	Gel electrophoresis; naked eye (turbidity)	10 fg	N/A	100% (8)	100% (17)	Milk	Spiked	10^2^ CFU/mL	N/A	N/A	Zhu *et al.* (2008)
1, 5	2008	Japan	*Salmonella* O9 group	IS200/IS1351 gene	Loopamp realtime turbidimeter	Real-time turbidity	12 CFU	1000 ×	100% (128)	100% (284)	Chicken cecal dropping	Spiked	N/A	6.1 × 10^1–^6.1 × 10^4^ CFU/g	100% Agreement with culture except for one *in vivo* spiked sample	Okamura *et al.* (2008)
1, 3	2008	China	*Salmonella* spp.	*invA*	Unspecified	Gel electrophoresis	N/A	0.01 ×	N/A	N/A	Raw milk	Spiked	>10^8^ CFU/mL	N/A	N/A	Wang *et al.* (2008a)
2, 3	2009	China	*Salmonella* spp.	*invA*	*In situ* LAMP	Inverted fluorescence microscopy (Cy3)	10 CFU	N/A	100% (6)	100% (2)	Eggshell	Spiked	10 CFU	N/A	N/A	Ye *et al.*^2009^)
1	2009	Japan	*Salmonella* O4 group	*rfbJ*	Loopamp realtime turbidimeter	Real-time turbidity; gel electrophoresis	10^0^ CFU	100 ×	100% (55)	100% (74)	N/A	N/A	N/A	N/A	N/A	Okamura *et al.* (2009)
3	2009	Japan	*Salmonella* spp.	*invA*	Loopamp realtime turbidimeter	Real-time turbidity	N/A	N/A	100% (54)	100% (40)	Various food	Spiked	10^2^ CFU/mL	N/A	N/A	Ueda and Kuwabara (2009)
1	2009	China	*Salmonella* spp.	*invA*	EMA-LAMP	Naked eye (colorimetry-SYBR Green I)	100 fg	>1000 ×	N/A	N/A	N/A	N/A	N/A	N/A	N/A	Lu *et al*. (2009)
1, 3	2009	China	*Salmonella* spp.	*phoP*	Heat block	Naked eye (turbidity and colorimetry-SYBR Green I); gel electrophoresis	35 CFU	N/A	100% (66)	100% (73)	Minced pork and raw milk	Both	N/A	35 CFU/250 mL	100% Agreement with culture for spiked and natural samples	Li *et al.* (2009)
1	2010	Korea	*Salmonella* spp.	*invA*	Thermal cycler (GeneAmp 2700)	Gel electrophoresis	0.21 CFU	10,000 × , 10 × (Real-time PCR)	N/A	N/A	N/A	N/A	N/A	N/A	N/A	Ahn *et al.* (2010)
3	2010	United States	*Salmonella* spp.	*invA*	RT-LAMP	Naked eye (turbidity); gel electrophoresis	500 CFU (gel electrophoresis), 0.05 CFU (naked eye)	N/A	N/A	N/A	Pork	Both	10^6^ CFU/25 g	10^2^ CFU/25 g	100% Agreement with culture for pork carcass swab, more sensitive than culture in pork	Techathuvanan *et al.* (2010)
3	2010	China	*Salmonella* spp.	Unspecified	Water bath	Naked eye (colorimetry-SYBR Green I)	N/A	N/A	N/A	N/A	Raw meat and dairy product	Both	N/A	10^2^ CFU/mL	Superior than culture	He *et al.* (2010)
1, 3	2010	China	*Salmonella* Enteritidis	*sdfI*	Water bath	Naked eye (turbidity and colorimetry-SYBR Green I); gel electrophoresis	4 CFU	1 × (Real-time PCR)	100% (5)	100% (8)	Pork and chicken	Natural	N/A	N/A	100% Agreement with real-time PCR	Yang *et al.* (2010)
1	2010	China	*Salmonella* spp.	*invA*	Water bath, heat block	Naked eye (colorimetry and fluorescence-SYBR Green I); gel electrophoresis	100 CFU or 1 pg	100 ×	97.8% (225)	100% (28)	N/A	N/A	N/A	N/A	N/A	Zhao *et al.* (2010)
3	2011	United States	*Salmonella* spp.	*invA*	RT-LAMP	Naked eye (turbidity); gel electrophoresis	N/A	N/A	N/A	N/A	Pork carcass and environment	Both	10^6^ CFU/500 mL	10^1^ CFU/500 mL	Same sensitivity as culture and rt-RT-PCR with or without enrichment	Techathuvanan *et al.* (2011)
2, 3	2011	China	*Salmonella* spp.	*invA*	*In situ* LAMP	Inverted fluorescence microscopy (Cy3)	10 CFU	50 ×	N/A	100% (1)	Eggshell	Spiked	N/A	1 CFU/cm^2^	N/A	Ye *et al.* (2011)
1, 3	2011	United States	*Salmonella* spp.	*invA*	PMA-LAMP on Loopamp realtime turbidimeter (LA-320C)	Real-time turbidity; naked eye (colorimetry-SYBR Green I)	3.4–34 CFU	100 × , 1 × (Real-time PCR)	100% (28)	100% (25)	Produce (cantaloupe, spinach, and tomato)	Spiked	6.1 × 10^3–^6.1 × 10^4^ CFU/g	40 CFU/g	Comparable to PMA-real-time PCR	Chen *et al.* (2011)
1, 3	2011	China	*Salmonella* spp., Shigella spp.	*invA*, *ipaH*	Multiplex LAMP-RFLP	Naked eye (turbidity); gel electrophoresis; RFLP	100 fg	10 ×	100% (8)	100% (12)	Milk	Spiked	N/A	5 CFU/10 mL	N/A	Shao *et al.* (2011)
3	2011	United States	*Salmonella* spp.	*invA*	Thermal cycler (Bio-Rad)	Naked eye (fluorescence-calcein)	10^4^ CFU	0.01 × (Real-time PCR)	99% (191)	100% (48)	Produce	Spiked	N/A	2 CFU/25 g	100% Agreement with BAM, real-time PCR, and rt-RT-PCR	Zhang *et al.* (2011)
1, 2	2011	United States	*Salmonella* spp. and five other waterborne pathogens	*invA*, *phoB*	Microfluidic chip and film heater, real-time thermal cycler (Opticon)	CCD camera; real-time fluorescence (SYTO-82)	N/A	N/A	N/A	N/A	N/A	N/A	N/A	N/A	N/A	Ahmad *et al.* (2011)
1, 2, 3	2011	United States	*Salmonella* spp.	*invA*	Handheld device with assimilating probes	Real-time fluorescence (FAM)	76 fg	N/A	N/A	N/A	Chicken	Both	25 CFU	N/A	Comparable to real-time PCR without enrichment; agreeable with PCR and culture in a natural sample after enrichment	Jenkins *et al.* (2011)
1, 3	2012	China	*Salmonella* spp.	*fimY*	Loopamp realtime turbidimeter (LA-320C)	Real-time turbidity; naked eye (colorimetry-SYBR Green I)	13 CFU	10 ×	100% (81)	100% (20)	Deli meat (chicken, pork, beef, shrimp, and mutton)	Both	N/A	6.3 × 10^3^ CFU/5 g	100% Agreement with culture, superior than PCR	Zhang *et al.* (2012b)
1, 5	2012	China	*Salmonella* spp.	*fimY*	Unspecified	Gel electrophoresis; naked eye (colorimetry-SYBR Green I)	4.8–6 CFU	10 ×	100% (86)	100% (23)	Duck organ	Both	6 CFU	N/A	100% Agreement with culture, superior than PCR	Tang *et al.* (2012)
3	2012	United States	*Salmonella* spp.	*invA*	RT-LAMP	Gel electrophoresis	5 × 10^4^ CFU	N/A	N/A	N/A	Liquid whole eggs	Both	10^8^ CFU/25 mL	10^0^ CFU/25 mL	Higher sensitivity in culture	Techathuvanan and D'Souza (2012)
2	2012	United States	*Salmonella* spp.	*invA*	Microfluidic chip and heat block	Electrochemical reporter (methylene blue); gel electrophoresis	16 CFU	N/A	N/A	100% (2)	N/A	N/A	N/A	N/A	N/A	Hsieh *et al.* (2012)
1, 3	2012	Iran	*Salmonella* serogroup D	*prt* (*rfbS*)	Thermal cycler (Veriti), water bath	Naked eye (turbidity); gel electrophoresis	10 CFU	10 ×	100% (5)	100% (4)	Chicken meat	Spiked	N/A	1–5 CFU/250 mL	Superior performance than PCR	Ravan and Yazdanparast (2012b)
1, 3	2012	China	*Salmonella* spp.	*hisJ*	Unspecified	Naked eye (turbidity and colorimetry-SYBR Green I); gel electrophoresis	16 CFU	10 ×	100% (79)	100% (23)	Pork, chicken, and vegetable	Natural	N/A	N/A	29 Out of 200 samples were positive by LAMP, 27 positive by PCR, and 34 positive by culture	Zhang *et al.* (2012a)
2, 3	2012	Iran	*Salmonella* serogroup D	*prt* (*rfbS*)	LAMP-ELISA	ELISA; gel electrophoresis	4 CFU	10 ×	100% (5)	100% (4)	Meat	Spiked	10^3^ CFU/mL	10 CFU/mL	Shorter enrichment needed compared to PCR-ELISA	Ravan and Yazdanparast (2012a)
1, 3	2012	China	*Salmonella* spp., Shigella spp., *Staphylococcus aureus*	*invA*	Multiplex LAMP-sequencing	Naked eye (turbidity); gel electrophoresis	10 fg	10,000 ×	100% (14)	100% (19)	Milk, pork, egg, and chicken	Natural	N/A	N/A	100% Agreement with culture and PCR	Jiang *et al.* (2012)
2	2012	United States	*Salmonella* spp., *Campylobacter jejuni*, *Shigella*, *Vibrio cholerae*	*invA*, *phoP*	Microfluidic chip and chip cartridge	Real-time fluorescence (SYTO-82)	10 CFU (*invA*), 100 CFU (*phoP*)	N/A	N/A	N/A	N/A	N/A	N/A	N/A	N/A	Tourlousse *et al.* (2012)
6	2012	Greece	*Salmonella* spp.	*invA*	Thermal cycler (MJ Mini)	Gel electrophoresis; naked eye (colorimetry and fluorescence)	N/A	N/A	100% (50)	100% (10)	N/A	N/A	N/A	N/A	N/A	Ziros *et al.* (2012)
1, 3	2013	China	*Salmonella* spp.	*invA*	Unspecified	Gel electrophoresis	N/A	N/A	100% (7)	100% (13)	Raw milk	Both	6–9 CFU	N/A	Without enrichment, 89.58% concordance with ISO 6579, 100% concordance with enrichment	Wang and Wang (2013)
3	2013	Italy	*Salmonella* spp.	*invA*	3M MDS (prototype)	Real-time bioluminescence	N/A	N/A	N/A	N/A	Retail meat (fresh and prepared)	Natural	N/A	<0.3–2.1 MPN/g	78.9% for LAMP and 90.5% for ISO 6579	Bonardi *et al.* (2013)
2, 3	2013	United States	*Salmonella* spp.	*invA*	Noninstrumented nucleic acid amplification (NINA) device (Thermos bottle)	Endpoint fluorescence (FAM)	92 fg	N/A	N/A	N/A	Milk	Spiked	2.8 × 10^4^ CFU/mL	1.4 CFU/mL	N/A	Kubota *et al.* (2013)
1, 2, 5	2013	United States	*Salmonella* spp.	*recF*	IMED chip and E-DNA sensor	E-DNA sensor (methylene blue)	N/A	N/A	N/A	N/A	Whole blood of mice	Natural	800 CFU/mL	N/A	N/A	Patterson *et al.* (2013)
1, 3	2013	Korea	*Salmonella* spp.	*invA*	OptiGene Genie II	Real-time fluorescence	3.2 CFU	100 ×	100% (56)	100% (12)	Duck carcass	Both	3.2 × 10^3^ CFU/mL	3.2 CFU/mL	96% sensitivity compared to culture, while PCR had 52% sensitivity	Cho *et al.* (2013)
2	2013	United States	*Salmonella* spp., *Escherichia coli* O157, *Listeria monocytogenes*	*invA*	Microfluidic chip and heater	Real-time fluorescence (EvaGreen)	N/A	N/A	N/A	N/A	N/A	N/A	N/A	N/A	N/A	Duarte *et al.* (2013)
3	2013	United States	*Salmonella* spp.	*invA*	Loopamp realtime turbidimeter (LA-320C)	Real-time turbidity	1 CFU	100 ×	100% (33)	N/A	Shell egg	Spiked	10^4^ CFU/25 mL	10^0^ CFU/25 mL	Shorter enrichment needed compared to PCR	Yang *et al.* (2013)
3, 4	2013	United States	*Salmonella* spp.	*invA*	3M MDS	Real-time bioluminescence	N/A	N/A	N/A	N/A	Ground beef and wet dog food	Spiked	N/A	0.72 CFU/375 g	No significant difference in the number of positive samples compared to USDA or FDA reference methods	Bird *et al.* (2013)
6	2013	Papua New Guinea	*Salmonella* spp., *Shigella* spp., *V. cholerae*	*phoP*	Loopamp endpoint turbidimeter	Naked eye (turbidity and colorimetry-HNB and SYBR Green I; endpoint turbidity	48 CFU	0.1 × (Real-time PCR)	N/A	N/A	N/A	N/A	N/A	N/A	N/A	Soli *et al.* (2013)
1, 3	2014	India	*Salmonella* Typhimurium	*typh*	Unspecified	Naked eye (turbidity and colorimetry-SYBR Green I); gel electrophoresis	2 pg	100 ×	100% (28)	100% (28)	Chicken meat	Natural	N/A	N/A	100% Agreement with culture and PCR	Kumar *et al.* (2014)
3	2014	United States	*Salmonella* spp.	*invA*	Loopamp realtime turbidimeter (LA-320C)	Real-time turbidity	N/A	N/A	N/A	N/A	Meat, chicken, egg, peanut butter, and produce	Spiked	N/A	N/A	More robust than PCR or real-time PCR for food applications	Yang *et al.* (2014)
2	2014	United States	*Salmonella* spp.	*invA*	UDG-LAMP	Naked eye (colorimetry and fluorescence-calcein); gel electrophoresis	4 × 10^4^ CFU	N/A	N/A	N/A	N/A	N/A	N/A	N/A	N/A	Hsieh *et al.* (2014)
3	2014	United States	*Salmonella* spp.	*invA*	Loopamp realtime turbidimeter (LA-500)	Real-time turbidity	N/A	N/A	100% (100)	100% (30)	Meat and produce	Spiked	N/A	1 CFU/test portion	100% Agreement	Bapanpally *et al.* (2014)
6	2014	South Africa	*Salmonella* spp., *Listeria*, *E. coli* O157:H7	*invA*	3M MDS	Real-time bioluminescence	N/A	N/A	N/A	N/A	Wastewater and river water	Natural	N/A	N/A	8 Samples positive by LAMP in contrast to 24 samples positive by PCR (different DNA extracts were used)	Loff *et al.* (2014)
1	2014	China	*Salmonella* spp., *E. coli* O157, *Listeria*, *Pseudomonas aeruginosa*, *Vibrio parahaemolyticus*	*invA*	Unspecified	Unspecified	N/A	N/A	100% (40)	100% (22)	N/A	N/A	N/A	N/A	N/A	Deng *et al.* (2014)
3, 4	2014	United States	*Salmonella* spp.	*invA*	3M MDS	Real-time bioluminescence	N/A	N/A	N/A	N/A	Ground beef and wet dog food	Spiked	N/A	0.72 CFU/375 g	No significant difference in the number of positive samples compared to USDA or FDA reference methods	Bird *et al.* (2014)
1, 5	2014	China	*Salmonella* spp.	*bcfD*	Loopamp realtime turbidimeter (LA-500)	Real-time turbidity; gel electrophoresis	5 CFU	10 ×	100% (44)	100% (9)	Chicken feces	Both	5 × 10^3^ CFU/g	N/A	N/A	Zhuang *et al.* (2014)
2	2015	United States	*Salmonella* spp., *Ralstonia solanacearum*	*invA*	Duplex LAMP on real-time thermal cycler (iQ5)	Real-time fluorescence (FAM and TAMRA)	500 fg (98 CFU) singleplex and 50 pg (9.8 × 10^3^ CFU) duplex	N/A	N/A	N/A	N/A	N/A	N/A	N/A	N/A	Kubota and Jenkins (2015)
1, 2	2015	Malaysia	*Salmonella* spp.	*fadA*	Microfluidic CD and heater	Naked eye (colorimetry-SYBR Green I); electrochemical sensor-SYBR Green I	6.25 pg, 85 CFU	100 ×	N/A	N/A	N/A	N/A	N/A	N/A	N/A	Uddin *et al.* (2015)
2, 3	2015	Denmark	*Salmonella* spp.	*invA*	Microfluidic chip and heater	Real-time fluorescence (SYTO-62); gel electrophoresis	N/A	N/A	N/A	N/A	Pork	Spiked	50 CFU/test	N/A	Similar sensitivity as conventional PCR	Sun *et al.* (2015)
3	2015	United States	*Salmonella* spp.	*invA*	Loopamp realtime turbidimeter (LA-320C)	Real-time turbidity	1.8–4 CFU	1–10 × (Real-time PCR)	100% (151)	100% (27)	Produce (cantaloupe, pepper, lettuce, sprout, and tomato)	Spiked	10^4–^10^6^ CFU/25 g	1.1–2.9 CFU/25 g	For several serovars, real-time PCR required higher cell concentration or longer enrichment time	Yang *et al.* (2015)
1, 3	2015	Thailand	*Salmonella* spp.	*stn*	Unspecified	Naked eye (turbidity and colorimetry-SYBR Green I); gel electrophoresis	5 fg, 1 CFU	N/A	100% (102)	100% (57)	Pork, chicken, and vegetables	Both	220 CFU/g	2 CFU/g	100% Agreement with BAM culture	Srisawat and Panbangred (2015)
6	2015	United States	*Salmonella* spp., *L. monocytogenes*, *S. aureus*, STEC, *Streptococcus agalactiae*	*invA*	Real-time thermal cycler (Applied Biosystems StepOne)	Real-time fluorescence	1 pg	N/A	N/A	N/A	N/A	N/A	N/A	N/A	N/A	Wang *et al.* (2015a)
1, 3	2015	China	*Salmonella* spp., *Salmonella* Choleraesuis, *Salmonella* Enteritidis, and *Salmonella* Typhimurium	*invE*, *fliC*, *lygD*, STM4495	Thermal cycler (Whatman Biometra UNO II)	Gel electrophoresis; naked eye (fluorescence-SYRR Green)	13.3–20 CFU/mL	10–100 ×	100% (3)	100% (7)	Pork	Spiked	16.7–26.7 CFU/mL	N/A	N/A	Chen *et al.* (2015)
2	2015	United States	*Salmonella* spp., *E. coli*, viruses, human sequences	*invA*	LAMP-PiBA	Optical detection of PiBA using a cell phone	N/A	N/A	N/A	N/A	N/A	N/A	N/A	N/A	N/A	DuVall *et al.* (2015)
3	2015	Singapore	*Salmonella* spp.	*invA*	3M MDS	Real-time bioluminescence	N/A	N/A	N/A	N/A	Duck wing, mung bean sprout, and fishball	Both	N/A	10^0^ CFU/25 g	20% Sensitivity in spiked samples, 91% sensitivity in natural samples	Lim *et al.* (2015)
3	2015	Greece	*Salmonella* spp., *L. monocytogenes*	*invA*	Real-time thermal cycler (Roche LightCycler Nano)	Real-time fluorescence; gel electrophoresis; naked eye (fluorescence-SYBR Green I)	N/A	N/A	N/A	100% (3)	RTE produce	Spiked	2 × 10^4^–1 × 10^7^ CFU/g	1–3 CFU/g	N/A	Birmpa *et al.* (2015a)
3	2015	United States	*Salmonella* spp.	*invA*	Real-time thermal cycler (MJ DNA Engine Opticon 2)	Real-time fluorescence (Midori Green); endpoint turbidity; gel electrophoresis	4 CFU	N/A	N/A	N/A	Lettuce	Spiked	4 CFU/g (10 CFU/reaction)	N/A	N/A	Wu and Levin (2015)
2, 3	2015	Greece	*Salmonella* spp., *L. monocytogenes*, adenovirus	*invA*	Custom-made LAMP platform	Real-time fluorescence; gel electrophoresis; naked eye (fluorescence-SYBR Green I)	N/A	N/A	N/A	N/A	RTE produce	Spiked	10^6^–10^7^ CFU/g	N/A	N/A	Birmpa *et al.* (2015b)
6	2015	United States	*Salmonella* spp.	*invA*	Real-time thermal cycler (MJ DNA Engine Opticon 2)	Real-time fluorescence (Midori Green); gel electrophoresis	7 CFU	N/A	N/A	N/A	N/A	N/A	N/A	N/A	N/A	Wu *et al.* (2015a)
3	2015	China	*Salmonella* spp.	*invA*	EMA-LAMP and PMA-LAMP on real-time thermal cycler (MJ DNA Engine Opticon 2)	Real-time fluorescence (Midori Green); endpoint turbidity; gel electrophoresis	N/A	N/A	N/A	N/A	Lettuce	Spiked	25 CFU/50 g (6 CFU/reaction)	N/A	N/A	Wu *et al.* (2015b)
4	2015	United Kingdom	*Salmonella* spp.	*invA*	Duplex LAMP on OptiGene Genie II	Real-time fluorescence	3.3 × 10^4^ CFU	N/A	N/A	N/A	Animal feed ingredient	Both	N/A	N/A	100% Agreement with ISO 6579:2002	D'Agostino *et al.* (2015)
6	2015	Poland	*Salmonella* spp.	*invA*	Unspecified	Gel electrophoresis	N/A	N/A	N/A	N/A	N/A	N/A	N/A	N/A	N/A	Futoma-Koloch *et al.* (2015)
1, 3	2015	China	*Salmonella* spp., *Shigella* spp.	*invA*	Loopamp realtime turbidimeter (LA-320C)	Real-time fluorescence (HEX); naked eye (colorimetry-calcein); gel electrophoresis	125 fg	100 × , 10 × (Real-time PCR)	100% (15)	100% (39)	Milk	Spiked	3.2 × 10^2^ CFU/mL	N/A	10 × (Real-time PCR), 100 × (PCR)	Wang *et al.* (2015c)
1, 2	2016	Korea	*Salmonella* spp., *E. coli* O157:H7, *V. parahaemolyticus*	*serA*	Microfluidic device (centrifugal) and lab oven	Naked eye (EBT); UV-Vis spectrophotometry	N/A	N/A	N/A	N/A	N/A	N/A	N/A	N/A	N/A	Oh *et al.* (2016b)
1	2016	China	*Salmonella* spp., *L. monocytogenes*	*invA*	Unspecified	Naked eye (colorimetry and fluorescence); gel electrophoresis	200 CFU	100 ×	100% (4)	100% (7)	N/A	N/A	N/A	N/A	N/A	Xiong *et al.* (2016)
2, 3	2016	Canada	*Salmonella* Enteritidis	*sdfI*	LAMP-SERS	SERS; gel electrophoresis	0.132 CFU	100 ×	100% (4)	100% (5)	Milk	Spiked	6 × 10^3^ CFU/mL	N/A	N/A	Draz and Lu (2016)
1, 3	2016	China	*Salmonella* spp.	gene62181533	Unspecified	Naked eye (turbidity and colorimetry-calcein); gel electrophoresis	1.586 CFU, 11.52 fg	100–10,000 ×	100% (32)	100% (25)	Milk and meat	Both	N/A	0.81 CFU/mL	For spiked samples, similar to culture methods; for natural samples, 100% agreement with culture and PCR	Li *et al.* (2016)
1, 5	2016	China	*Salmonella* Enteritidis, *Salmonella* Gallinarum	*sefA*	Loopamp realtime turbidimeter (LA-500)	Real-time turbidity; gel electrophoresis	4 CFU	10 ×	100% (163)	100% (14)	Chicken feces	Spiked	400 CFU	N/A	More sensitive than culture, but statistically insignificant	Gong *et al.* (2016)
2	2016	Spain	*Salmonella* spp., bovine species	*invA*	In-disc LAMP (iD-LAMP)	Naked eye (turbidity-direct and PEI); real-time colorimetry-HNB	5 CFU	N/A	100% (7)	100% (4)	N/A	N/A	N/A	N/A	1 × (Conventional LAMP)	Santiago-Felipe *et al.* (2016)
1, 2, 3	2016	Korea	*Salmonella* spp., *E. coli* O157:H7, *L. monocytogenes*, *V. parahaemolyticus*	*invA*	Microfluidic device (centrifugal) and miniaturized rotary instrument with heat blocks	Naked eye (colorimetry-EBT); UV-Vis spectrophotometry	N/A	N/A	N/A	N/A	Milk	Spiked	N/A	N/A	N/A	Oh *et al.* (2016a)
1, 2, 3	2016	Malaysia	*Salmonella* spp.	*invA*	Microfluidic CD and hot air gun	Naked eye (colorimetry-SYBR Green I)	12.5 pg	N/A	N/A	100% (6)	Tomato	Spiked	3.4 × 10^4^ CFU/mL	N/A	100 × (PCR), 1 × (conventional LAMP)	Sayad *et al.* (2016)
2	2016	China	*Salmonella* spp., *Bacillus cereus*, *E. coli*, *Vibrio fluvialis*, *V. parahaemolyticus*	*invA*	Microfluidic chip (SlipChip) and custom heater	Naked eye (fluorescence-calcein); CCD camera; inverted fluorescence microscope; gel electrophoresis	N/A	N/A	N/A	N/A	N/A	N/A	N/A	N/A	N/A	Xia *et al.* (2016)
1, 2, 3, 4	2016	United States	*Salmonella* spp.	*invA*	3M MDS	Real-time bioluminescence	36 CFU	N/A	100% (151)	100% (27)	Food and feed	Spiked	10^4^–10^6^ CFU/25 g	1–3 CFU/25 g	N/A	Yang *et al.* (2016)
3	2016	United States	*Salmonella* spp.	*invA*	3M MDS	Real-time bioluminescence	N/A	N/A	N/A	N/A	Ground beef and peanut butter	Spiked	N/A	0.67 CFU/325 g	No significant difference in the number of positive samples compared to USDA or FDA reference methods	Bird *et al.* (2016)
1, 5	2016	India	*Salmonella* spp.	*invA*	Real-time thermal cycler (Agilent Mx3000P)	Real-time fluorescence; naked eye (turbidity, colorimetry, and fluorescence-SYBR Green I)	10 CFU	10 × (Real-time PCR)	100% (12)	100% (15)	Fecal sample	Natural	N/A	N/A	Higher sensitivity than real-time PCR, but statistically insignificant	Mashooq *et al.* (2016)
3	2016	Poland	*Salmonella* spp.	*invA*	3M MDS	Real-time bioluminescence	N/A	N/A	N/A	N/A	Various food	Natural	N/A	N/A	100% Agreement with ISO culture method	Sarowska *et al.* (2016)
4	2016	United Kingdom	*Salmonella* spp.	*invA*	Duplex LAMP on OptiGene Genie II	Real-time fluorescence	N/A	N/A	99% (100)	100% (30)	Animal feed ingredient (soya meal)	Spiked	N/A	1 CFU/100 g	Full agreement (RLOD of 1) with ISO 6579 culture method	D'Agostino *et al.* (2016)
3	2016	Malaysia	*Salmonella* spp.	*invA*	3M MDS	Real-time bioluminescence	N/A	N/A	N/A	N/A	Poultry and processing environment	Natural	N/A	N/A	Substantial agreement with ISO culture method	Abirami *et al.* (2016)
2	2017	China	*Salmonella* spp., *E. coli*, *L. monocytogenes*, *P. aeruginosa*, *V. parahaemolyticus*	*invA*	Colony LAMP	Naked eye (colorimetry-SYBR Green I); gel electrophoresis	100 CFU	100–1000 ×	100% (15)	100% (101)	N/A	N/A	N/A	N/A	N/A	Yan *et al.* (2017)
1, 3	2017	Korea	*Salmonella* spp.	*invA*	PMA-LAMP on OptiGene Genie II	Real-time fluorescence	80 CFU	10 ×	100% (140)	100% (27)	Chicken carcass rinse	Spiked	1 × 10^3^ CFU/mL	N/A	N/A	Youn *et al.* (2017)
2, 5	2017	China	*Salmonella* spp., *E. coli*, *Proteus hauseri*, *V. parahaemolyticus*	*invA*	In-gel LAMP (gLAMP)	Inverted fluorescence microscopy-calcein	2 CFU/μL	N/A	N/A	100% (3)	Human serum	Spiked	1.3 × 10^4^ CFU/mL	N/A	N/A	Chen *et al.* (2017)
1, 2	2017	Korea	*Salmonella* spp., *E. coli* O157:H7, *S. aureus*	*invE*	CMOS integrated system	Real-time photon count-HNB; gel electrophoresis	N/A	N/A	N/A	N/A	N/A	N/A	N/A	N/A	N/A	Wang *et al.* (2017)
6	2017	China	*Salmonella* spp., *V. parahaemolyticus*	*bcfD*	Duplex LAMP	Real-time fluorescence	20 pg	1 ×	100% (7)	100% (12)	N/A	N/A	N/A	N/A	N/A	Liu *et al.* (2017)
1, 2, 3	2017	China	*Salmonella* spp.	*invA*	DNAzyme LAMP (dLAMP)	Naked eye (colorimetry-DNAzyme); gel electrophoresis	0.5 pg	N/A	N/A	100% (2)	Pork	Spiked	N/A	N/A	N/A	Zhu *et al.* (2017)
3	2017	United States	*Salmonella* spp.	*invA*	3M MDS	Real-time bioluminescence	N/A	N/A	N/A	N/A	Egg products (20 types)	Spiked	N/A	1.63–4.18 CFU/25 g	Complete agreement with BAM culture and ANSR	Hu *et al.* (2017)
2, 3	2017	Korea	*Salmonella* spp., *V. parahaemolyticus*	*invA*	Integrated rotary microfluidic system	Laternal flow strip	50 CFU	N/A	N/A	N/A	Milk	Spiked	10^4^ CFU/mL	N/A	N/A	Park *et al.* (2017)
1, 2, 3	2017	China	*Salmonella* spp.	*siiA*	LAMP-LFD	LFD; gel electrophoresis	7.4 × 10^−3^ CFU	100 ×	100% (21)	100% (31)	Powdered infant formula	Spiked	2.2 CFU/g	N/A	100% Accuracy	Zhao *et al.* (2017)
2	2017	Korea	*Salmonella* spp., *E. coli* O157:H7, *V. parahaemolyticus*	*invA*	Microfluidic device (centrifugal) and lab oven	Naked eye (colorimetry-EBT); RGB-based image processing	500 CFU	N/A	N/A	N/A	N/A	N/A	N/A	N/A	N/A	Seo *et al.* (2017)
1, 3	2017	Portugal	*Salmonella* spp., *Salmonella* Enteritidis, *Salmonella* Typhimurium	*invA*, *safA*, STM4497	Real-time thermal cycler (Applied Biosystems StepOne)	Real-time fluorescence (Midori Green)	0.32 ng (5.6 ng for *safA*)	10 × (0.1 × for *safA*; real-time PCR)	100% (12)	100% (12)	Poultry and eggs	Spiked	N/A	4–10 CFU/25 g	>97% Agreement with culture	Garrido-Maestu *et al.* (2017b)
2, 3	2017	Portugal	*Salmonella* spp.	*invA*	Microfluidic chip and incubator	Naked eye (colorimetry-AuNP); gel electrophoresis	N/A	N/A	N/A	N/A	Chicken, turkey, and eggs	Spiked	N/A	10 CFU/25 g	100% Agreement with culture	Garrido-Maestu *et al.* (2017a)
4	2018	United States	*Salmonella* spp.	*invA*	OptiGene Genie II, loopamp realtime turbidimeter (LA-500)	Real-time fluorescence; real-time turbidity	1.3–28 CFU	1 × (Real-time PCR)	100% (247)	100% (53)	Animal feed and pet food	Spiked	N/A	0.0062 MPN/g	Combined RLOD of 0.61	Domesle *et al.* (2018)
2	2018	China	*Salmonella* spp., *P. aeruginosa*, *Streptococcus iniae*, *Vibrio alginolyticus*, *V. parahaemolyticus*, *Vibrio vulnificus*	*invA*	Microfluidic device (hand-powered centrifugal) and pocket warmers	Real-time fluorescence; gel electrophoresis	2 × 10^4^ CFU/μL	N/A	N/A	N/A	N/A	N/A	N/A	N/A	N/A	Zhang *et al.* (2018)
1, 2, 3	2018	Malaysia	*Salmonella* spp., *E. coli*, *V. cholerae*	Unspecified	Microfluidic device (centrifugal)	Naked eye (colorimetry-calcein)	N/A	100 ×	100% (8)	100% (20)	Chicken meat	Spiked	30 fg/μL	N/A	N/A	Sayad *et al.* (2018)
1, 3	2018	United States	*Salmonella* Enteritidis	*prot6E*	OptiGene Genie III	Naked eye (colorimetry-calcein)	1.2–12 CFU	1 ×	97.4% (114)	100% (69)	Egg products (22 types)	Spiked	N/A	1–5 CFU/25 g	100% Agreement with BAM and real-time PCR	Hu *et al.* (2018)
2, 3	2018	Greece	*Salmonella* spp.	*invA*	Integrated micro-nano-bio acoustic system	Surface acoustic wave sensor; gel electrophoresis	2 CFU	N/A	N/A	N/A	Milk	Spiked	N/A	1 CFU/25 mL	N/A	Papadakis *et al.* (2018)
1, 3	2018	China	*Salmonella* spp.	*invA*	PMA-LAMP on heat block, real-time thermal cycler (CFX96)	Naked eye (colorimetry-calcein); real-time fluorescence (calcein)	1.6 CFU	1 × (Real-time PCR)	100% (3)	100% (28)	Eggs, tomato, cucumber, lettuce, dried squid, skim milk powder, and meat	Both	6.3×10^3^ CFU/mL	6.3×10^1^ CFU/mL	100% Agreement with BAM and real-time PCR	Fang *et al.* (2018)

^a^ Studies focusing on assay development (1), platform development (2), application in food (3), application in feed (4), application in clinical samples (5), and other developments/applications (6).

^b^ When authors were from multiple countries, only the corresponding author's country is listed.

^c^ By default, the sensitivity (limit of detection) comparison was made to PCR unless specified otherwise.

ANSR, amplified nucleic single temperature reaction; AuNP, gold nanoparticle; BAM, FDA's Bacteriological Analytical Manual; CCD, charge-coupled device; CFU, colony-forming unit; CMOS, Complementary metal-oxide-semiconductor; EBT, Eriochrome Black T; E-DNA, Electrochemical DNA; ELISA, enzyme-linked immunosorbent assay; EMA-LAMP, ethidium monoazide loop-mediated isothermal amplification; HNB, hydroxy naphthol blue; IMED, integrated microfluidic electrochemical DNA; LAMP, loop-mediated isothermal amplification; LFD, lateral flow dipstick; MDS, Molecular Detection System; MPN, most probable number; PCR, polymerase chain reaction; PEI, polyethylenimine; PiBA, product-inhibited bead aggregation; PMA, propidium monoazide; RFLP, restriction fragment length polymorphism; RGB, red green blue; RLOD, relative level of detection; RTE, ready to eat; RT-LAMP, reverse transcriptase-LAMP; rt-RT-PCR, real-time reverse transcriptase PCR; SERS, surface-enhanced Raman spectroscopy; STEC, Shiga toxin–producing *E. coli*; UDG-LAMP, Uracil-DNA-glycosylase-supplemented LAMP; UV-Vis, ultraviolet and visible.

### Primer design

LAMP primers are commonly designed using the free web-based PrimerExplorer V4 software (V5 is available as of October 2016; http://primerexplorer.jp/e; Fujitsu Ltd., Tokyo, Japan). The LAMP Designer software (PREMIER Biosoft International, Palo Alto, CA) has been developed to serve a similar purpose. Each LAMP primer set contains four primers, two inner primers (FIP, forward inner primer; BIP, backward inner primer) and two outer primers (F3; B3). The inner primers FIP/BIP consist of complementary sequences of F1c/B1c and F2/B2 regions (Eiken Chemical Co. Ltd., 2009).

**FIG. 2.**
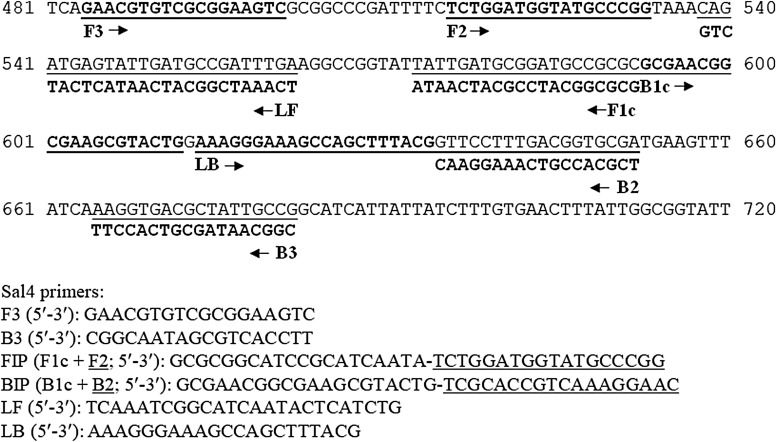
A sequence alignment to illustrate the positions of six LAMP primers (F3, B3, FIP, BIP, LF, and LB) on the target gene. Partial nucleotide sequence of the *Salmonella* invasion gene *invA* (GenBank accession No. M90846) is shown, which was the target gene used to design our *Salmonella* LAMP assay (Yang *et al.*, 2016). F3 and B3 are the forward and backward outer primers, respectively. FIP/BIP consists of complementary sequences of F1c/B1c and F2/B2 regions. BIP, backward inner primer; FIP, forward inner primer; LAMP, loop-mediated isothermal amplification; LB, loop backward; LF, loop forward.

In earlier *Salmonella* LAMP studies, a TTTT linker was often added to connect F1c and F2 or B1c and B2 (Wang *et al.*, 2008a; Lu *et al.*, 2009; Zhang *et al.*, 2012). It is now common practice for *Salmonella* LAMP assays to incorporate two loop primers (LF, loop forward; LB, loop backward) to accelerate the reaction (Nagamine *et al.*, 2002). Figure 2 illustrates the positions of these primers (or components of FIP/BIP) on the target gene, *invA*, which we used for a *Salmonella* LAMP assay (Yang *et al.*, 2016).

The *invA* gene is the most frequently targeted gene for designing LAMP primers for *Salmonella* spp. (74% of articles in Table 2). This gene is 2176 bp long in *Salmonella* Typhimurium (GenBank accession No. M90846) (Galan *et al.*, 1992). A closer examination of the regions (5′ end of F3 and 3′ end of B3) covered by the primers designed by Hara-Kudo *et al.* (2005) and us (Yang *et al.*, 2016) showed that they are in tandem with each other (225–468 and 484–682 bp), both overlapping with the region (371–655 bp) targeted by a set of widely used *Salmonella invA* PCR primers (Rahn *et al.*, 1992). Sequence analysis showed that other sets of *invA*-based LAMP primers also overlapped with this PCR region (Chen *et al.*, 2011), while still others targeted downstream regions (Wang *et al.*, 2008b; Shao *et al.*, 2011).

Other target genes, including *bcfD* and *fimY*, have also been used to design *Salmonella* LAMP primers (Table 2). *Salmonella* LAMP detection kits with proprietary primer information are available commercially, including Loopamp *Salmonella* Detection Kit (Eiken Chemical Co., Ltd., Tokyo, Japan), 3M Molecular Detection Assay (MDA) 2—*Salmonella* (3M Food Safety, St. Paul, MN), SAS Molecular Tests *Salmonella* Detection Kit (SA Scientific Ltd., San Antonio, TX), and Ampli-LAMP *Salmonella* species (NovaZym, Poznań, Poland).

A few LAMP assays have been developed that target specific *Salmonella* serovars or serogroups (Table 2). For instance, *sdfI* (Yang *et al.*, 2010) and *prot6E* (Hu *et al.*, 2018) were used to design two separate LAMP assays for *Salmonella enterica* serovar Enteritidis, while *typh* was used to specifically detect *Salmonella* Typhimurium (Kumar *et al.*, 2014). The *sefA* gene has been explored to design a LAMP assay for both *Salmonella* Enteritidis and *Salmonella* Gallinarum (Gong *et al.*, 2016). An insertion element IS200/IS1351 gene was used to detect *Salmonella* O9 serogroup (Okamura *et al.*, 2008), *prt* (*rfbS*) for serogroup D (i.e., O9) (Ravan and Yazdanparast 2012a, b), and *rfbJ* for O4 serogroup (Okamura *et al.*, 2009).

### Platform development

LAMP amplicons can be detected through multiple platforms/methods, as reviewed by Zhang *et al.* (2014), including naked eye, gel electrophoresis, colorimetry, turbidity, fluorescence, bioluminescence, electrochemical sensors/chips, lateral flow dipstick (LFD), and enzyme-linked immunosorbent assay (ELISA). Among them, detection by turbidity derived from magnesium pyrophosphate formation (white precipitate) has been the cornerstone of the LAMP technology (Mori *et al.*, 2001).

Recently, we have seen explosive growth in the development and commercialization of LAMP-based microchips and microdevices for POC molecular diagnostics, many using optical and electrochemical methods (Safavieh *et al.*, 2016). Some platforms are geared toward endpoint detection, while others focus on real-time detection. Given the large amount of DNA (10–20 μg/25 μL reaction mix) generated in a LAMP run (Kokkinos *et al.*, 2014), platforms that allow closed-tube detection are highly recommended to prevent cross-contamination.

**FIG. 3.**
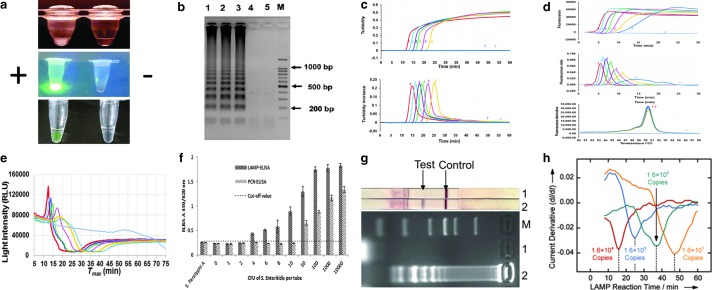
Monitoring methods used to detect LAMP amplicons. **(a)** Naked eye observation based on white precipitate (Hara-Kudo *et al.*, 2005), DNA dye (SYBR Green I) (Mashooq *et al.*, 2016), and colorimetric indictor (calcein) (Li *et al.*, 2016), respectively; **(b)** gel electrophoresis (Hara-Kudo *et al.*, 2005); **(c)** real-time turbidity (Domesle *et al.*, 2018); **(d)** real-time fluorescence (Domesle *et al.*, 2018); **(e)** BART (Yang *et al.*, 2016); **(f)** ELISA (Ravan and Yazdanparast, 2012); **(g)** LFD (Zhao *et al.*, 2017); and **(h)** electrochemical method (Hsieh *et al.*, 2012). BART, bioluminescent assay in real-time; ELISA, enzyme-linked immunosorbent assay; LAMP, loop-mediated isothermal amplification; LFD, lateral flow dipstick. Figure reprinted from Hsieh K, *et al.* 2012, Angewandte Chemie International Edition. Reproduced by permission of John Wiley & Sons, Inc.

As shown in Table 2, various platforms/methods have been developed for or adopted by *Salmonella* LAMP assays over the years. Figure 3 illustrates several examples of the monitoring methods used. In earlier studies, *Salmonella* LAMP reactions were run in water baths, heat blocks, or thermal cyclers, and detected by naked eye and gel electrophoresis (Table 2). Naked eye monitoring was generally performed in three ways (Zhang *et al.*, 2014): first by observing the white precipitate (turbidity) formed in a LAMP reaction tube (Fig. 3a, top), second by observing the color change postamplification after adding DNA-binding dyes such as SYBR Green I, either under normal air (colorimetry) or ultraviolet (fluorescence) (Fig. 3a, middle), and third by observing the color change or fluorescence in the LAMP reaction tube with metal indicators (e.g., calcein and hydroxy naphthol blue [HNB]) added during assay preparation (Fig. 3a, bottom). Gel electrophoresis was done postamplification by running an agarose gel and observing the characteristic ladder-like banding pattern of LAMP amplicons (Fig. 3b). Despite being widely used, concerns of introducing ambiguity (in the case of naked eye) or contamination (for gel electrophoresis) render these methods less desirable (Zhang *et al.*, 2014).

Real-time turbidity and real-time fluorescence have gained wide popularity as closed-tube or “one-pot” monitoring methods for *Salmonella* LAMP, especially with the recent availability of small, portable, robust, and user-friendly instruments (Fig. 1). As the LAMP reaction proceeds, turbidity or fluorescence readings are displayed in real time (amplification curves) and corresponding derivative values are plotted automatically at the completion of the run (derivative curves) (Fig. 3c, d). Results are interpreted based on whether these derivative values have reached thresholds set by the machine or user. While no modification to the LAMP reaction mix is needed for turbidity monitoring, to enable fluorescence detection, fluorophores are usually incorporated into the reaction mix or primers.

For turbidimetry-based *Salmonella* LAMP assays, Loopamp Realtime Turbidimeters LA-320 and LA-500 are commonly used platforms, whereas real-time PCR machines and Genie II have been used to develop several fluorescence-based *Salmonella* LAMP assays (Table 2). It is noteworthy that on the Genie II platform, an anneal step (from 98°C to 80°C with 0.05°C decrement per second) is included in each run to determine the annealing temperature of LAMP amplicons, which serves as an extra specificity check (Fig. 3d, bottom). Another closed-tube method used recently to monitor *Salmonella* LAMP reactions is based on bioluminescent assay in real time (BART) (Bird *et al.*, 2013, 2014, 2016; Yang *et al.*, 2016) (Fig. 3e) and performed in small platforms such as the 3M Molecular Detection System (MDS) (Fig. 1g). BART monitors the dynamic changes in the level of pyrophosphate produced in a LAMP reaction, which is converted to adenosine triphosphate (ATP) and utilized by firefly luciferase to emit light (Gandelman *et al.*, 2010).

Several platforms also pair *Salmonella* LAMP assays with other novel detection methods downstream. Referred to as “open-tube” reactions, the process involves transferring LAMP amplicons to a second tube or platform for endpoint detection. Ravan and Yazdanparast (2012a) developed a LAMP-ELISA to detect *Salmonella* serogroup D by generating digoxigenin-labeled LAMP amplicons followed by hybridization to serogroup-specific oligonucleotide probes coated on a microtiter plate and ELISA readout (Fig. 3f). Draz and Lu (2016) combined LAMP with surface-enhanced Raman spectroscopy (LAMP-SERS) for the specific detection of *Salmonella* Enteritidis. To enable SERS detection, LAMP amplicons were hybridized with Raman-active Au-nanoprobes followed by nuclease digestion and washes (Draz and Lu, 2016).

More recently, Zhao *et al.* (2017) explored LFD as a new detection method for *Salmonella* LAMP (LAMP-LFD) (Fig. 3g). The LAMP FIP and BIP primers were labeled at the 5′ end with biotin and fluorescein isothiocyanate (FITC), respectively. Gold nanoparticles conjugated with anti-FITC antibody were embedded in the conjugate pad during the LFD assembly, whereas streptavidin and anti-mouse secondary antibody were added on the detection region to form the test line and control line, respectively. LAMP amplicons were mixed with a running buffer followed by LFD immersion into the mixture for detection. Noticeably, these open-tube platforms require extensive postamplification manipulations, which are cumbersome, time-consuming, and prone to cross-contamination.

**FIG. 4.**
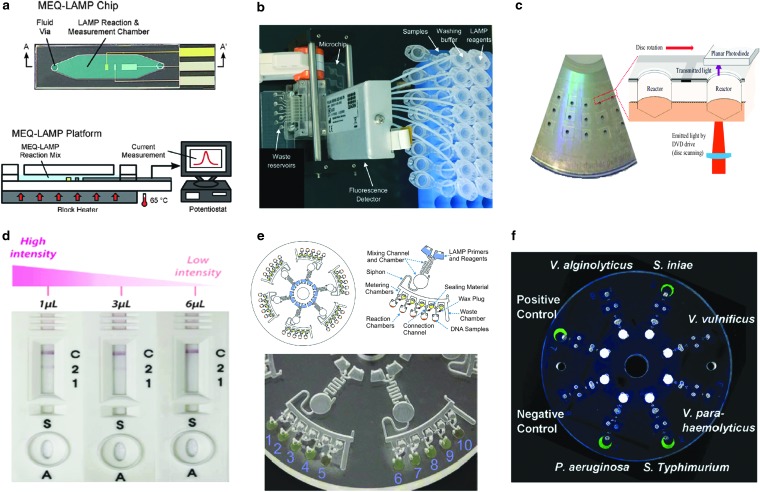
Microfluidic devices designed for LAMP-based detection of *Salmonella*. **(a)** MEQ-LAMP (Hsieh *et al.*, 2012); **(b)** eight-chamber LOC with integrated sample preparation (Sun *et al.*, 2015); **(c)** iD-LAMP (Santiago-Felipe *et al.*, 2016); **(d)** integrated rotary microfluidic LAMP (Park *et al.*, 2017); **(e)** centrifugal microfluidic LAMP (Sayad *et al.*, 2018); and **(f)** hand-powered centrifugal microfluidic LAMP (Zhang *et al.*, 2018). Figure reprinted in part from Hsieh K, *et al.* 2012, Angewandte Chemie International Edition. Reproduced with permission of John Wiley & Sons, Inc; and Sun Y, *et al.* 2015 and Zhang L, *et al.* 2018. Lab on a Chip. Reproduced with permission of The Royal Society of Chemistry. iD-LAMP, in-disc LAMP; LAMP, loop-mediated isothermal amplification; LOC, lab-on-a-chip; MEQ, microfluidic electrochemical quantitative.

Recently, there have been many LAMP-based microfluidic devices designed for POC and food applications; some have used *Salmonella* as the model organism to show proof of concept (Table 2). For instance, Hsieh *et al.* (2012) designed a microfluidic electrochemical quantitative LAMP (MEQ-LAMP) chip (Fig. 4a) that used integrated electrodes to monitor the intercalation of DNA binding dye methylene blue redox reporter molecules into LAMP amplicons in real time. LAMP amplification was correlated with a decrease in the measured current signals (shown in Fig. 3h). Sun *et al.* (2015) developed an eight-chamber lab-on-a-chip (LOC) system (Fig. 4b) with integrated magnetic bead-based sample preparation and parallel LAMP amplification for *Salmonella* detection in food. After evaluating several DNA binding dyes, SYTO-62 was chosen for on-chip real-time fluorescence detection. Santiago-Felipe *et al.* (2016) designed a compact disc microreactor for LAMP (in-disc LAMP, iD-LAMP) (Fig. 4c) and tested *Salmonella* as proof-of-concept; the reaction was monitored through HNB colorimetry.

Park *et al.* (2017) integrated DNA extraction, LAMP, and colorimetric lateral flow strip into a rotary microfluidic system (Fig. 4d) and demonstrated the parallel detection of *Salmonella* and *Vibrio parahaemolyticus* in milk. Very recently, Sayad *et al.* (2018) developed a centrifugal microfluidic platform (Fig. 4e) by incorporating a calcein-mediated colorimetric and wireless detection method for the parallel detection of *E. coli*, *Salmonella*, and *Vibrio cholerae* in food. Zhang *et al.* (2018) reported another centrifugal microfluidic platform (Fig. 4f) for parallel detection of six pathogens, *Salmonella* included, in a hand-powered, electricity-free format. The entire procedure, including nucleic acid purification, LAMP amplification, and visual detection of calcein-based fluorescence signals, is integrated into a microfluidic disc, achieving sample-to-result POC diagnostics (Zhang *et al.*, 2018).

### Assay optimization

Attempts to optimize LAMP reagent mix and/or reaction condition have been made in several *Salmonella* LAMP studies. Upon optimizing all components of a newly developed *Salmonella* LAMP assay, Chen *et al.* (2011) concluded that eliminating betaine from the LAMP reagent mix resulted in shorter time-to-positive results and stronger turbidity signals, that is, better amplification efficiency. In another study, the addition of betaine also contributed to a reduction in the amount of LAMP amplicons (Li *et al.*, 2016), whereas Garrido-Maestu *et al.*, (2017b) reported that with betaine, false positive results were generated from nontarget DNA as well as water. Instead, the addition of dimethyl sulfoxide (DMSO) at 7.5% was found to be favorable for LAMP amplification (Garrido-Maestu *et al.*, 2017b).

Multiple *Salmonella* LAMP studies have confirmed that the incorporation of loop primers significantly decreased the time taken to obtain positive results, often by 20 min or more (Okamura *et al.*, 2009; Zhuang *et al.*, 2014; Mashooq *et al.*, 2016). The reaction time for *Salmonella* LAMP assays ranges from 25 min to 2 h, and those requiring >60 min usually lacked loop primers (Ye *et al.*, 2011). Running temperatures for the assays fall between 60°C and 65°C, except that 66°C was used in three studies (Gong *et al.*, 2016; Park *et al.*, 2017; Seo *et al.*, 2017).

### Assay evaluation

Specificity (inclusivity and exclusivity) and sensitivity (pure culture/DNA and comparison with PCR) evaluations of newly developed *Salmonella* LAMP assays are usually performed at the time of initial assay development. Unfortunately, these key parameters are missing for quite a few studies, especially those focusing on proof-of-concept POC diagnostics. As shown in Table 2, the number of strains tested for inclusivity (range, 3–247) and exclusivity (range, 1–284) varies vastly among the studies. Many studies did not meet the recommendations of AOAC International (AOAC, 2012) and the International Organization for Standardization (ISO, 2016) on testing at least 100 *Salmonella* strains of different serovars for inclusivity and at least 30 competitive strains for exclusivity. Although strains belonging to *S. enterica* subsp. *enterica* (I) are well represented in inclusivity testing, those belonging to five other subspecies of *S. enterica* (i.e., *salamae* [II], *arizonae* [IIIa], *diarizonae* [IIIb], *houtenae* [IV], and *indica* [VI]) and *Salmonella bongori* are seldom tested. Nonetheless, almost all studies uniformly reported 100% inclusivity and 100% exclusivity for respective *Salmonella* LAMP assays developed, highlighting the highly specific nature of the LAMP technology.

Zhang *et al.* (2011) reported that one *S. enterica* subsp. *arizonae* strain CNM-247 and one *S. bongori* strain 95-0321 failed to be amplified by the Hara-Kudo's primer sets, neither did one *S. enterica* subsp. *arizonae* strain NCTC 7301 in another study (D'Agostino *et al.*, 2016), while successful amplification of seven *S. enterica* subsp. *arizonae* strains along with 220 *S. enterica* subsp. *enterica* strains of 39 serovars were shown at the time of assay development (Hara-Kudo *et al.*, 2005). Very recently, Domesle *et al.* (2018) evaluated the specificity of our *invA*-based *Salmonella* LAMP assay (Yang *et al.*, 2016) (Fig. 2) using 300 bacterial strains (247 *Salmonella* strains of 185 serovars and 53 non-*Salmonella* strains) and demonstrated 100% specificity on both turbidimetry- and fluorescence-based platforms. Eleven *S. enterica* subsp. *arizonae* strains were tested and when compared to those belonging to other *S. enterica* subspecies, significantly longer time-to-positive results were required for these *S. enterica* subsp. *arizonae* strains (Domesle *et al.*, 2018).

In pure-culture sensitivity testing, the reported limits of detection for all *Salmonella* LAMP assays ranged from 0.132 to 5 × 10^4^ colony-forming unit (CFU) per reaction with several reporting a level much lower than 1 CFU (Table 2). Among studies where genomic DNA was tested, the limits of detection fell between 5 fg and 5.6 ng per reaction (Table 2). These are equivalent to a range from 1 CFU to 1 × 10^6^ CFU per reaction, assuming one *Salmonella* genome weighs about 5 fg (Malorny *et al.*, 2004). Numerous studies also compared the sensitivity between LAMP and PCR or real-time PCR (Table 2). The superior performance of LAMP (10- to 10,000-fold better sensitivity) over PCR was observed in the majority of studies, while equal (Yang *et al.*, 2010; Liu *et al.*, 2017) or lower sensitivity (0.01-fold) of LAMP to PCR (Wang *et al.*, 2008a) was also reported. On the other hand, real-time PCR had limits of detection rather comparable (within 10-fold difference) to LAMP (Table 2).

## *Salmonella* LAMP Assay Application

Since 2008, the application of *Salmonella* LAMP assays in human food has expanded to numerous food matrices, such as chicken, turkey, pork, beef, produce, and milk. More recently, *Salmonella* LAMP assays have also been applied in animal food, that is, pet food, animal feed, and raw materials and ingredients (D'Agostino *et al.*, 2015; Bird *et al.*, 2016; Yang *et al.*, 2016). Below we present some challenges commonly associated with foodborne pathogen detection and the promise that LAMP offers and some actual applications.

### Challenges and promises

*Salmonella* detection in human and animal food faces many of the same inherent challenges associated with general food testing for pathogens (Ge and Meng, 2009; Wang *et al.*, 2013). Food and feed encompass many diverse and complex matrices, which presents a major hurdle toward developing effective sample preparation and testing strategies. Many matrices frequently harbor inhibitors to key reagents used in molecular assays, such as PCR enzymes, which greatly undermine the efficiency and utility of such assays. The presence of high levels of background flora in some matrices may also interfere with assay performance. Therefore, matrix-specific assay evaluations may be necessary. Furthermore, *Salmonella* is usually present in food or feed at much lower concentrations than those found in clinical specimens and the bacterial cells may be injured by the processes used to produce the food or feed (Ge and Meng, 2009).

To address these challenges, enrichment is commonly used to resuscitate injured *Salmonella* cells, increase the concentration of *Salmonella*, and dilute the effect of inhibitors and background flora on the assays (Wang *et al.*, 2013). This is a general strategy applied to improve pathogen detection in food and feed, which is not limited to LAMP.

One major advantage of LAMP over PCR is the high tolerance to biological substances, such as whole blood and urine, commonly found in clinical specimens (Kaneko *et al.*, 2007; Yang *et al.*, 2014). This advantage also translates into food testing for pathogens as a means to overcome matrix effects. We designed a study to specifically evaluate the robustness of a *Salmonella* LAMP assay for food applications (Yang *et al.*, 2014). Besides superior performance over PCR under abusive pH conditions, LAMP also showed greater tolerance to potential assay inhibitors (e.g., humic acid, soil, and culture media) than PCR. When food rinses, including meat juice, chicken rinse, egg homogenate, and produce homogenate, were added at 20% of the reaction mix, PCR amplifications were completely inhibited, but LAMP reactions were not (Yang *et al.*, 2014). The study highlights the promise of LAMP as a robust and powerful method for *Salmonella* detection in various food matrices.

### Application in food

As shown in Table 2, *Salmonella* LAMP assays have been applied in a wide variety of food matrices, including all the major food categories linked to *Salmonella* outbreak-associated illnesses, for example, produce, eggs, chicken, pork, and beef (IFSAC, 2015, 2017). The most widely adopted assay (in 27 studies) is the one developed by Hara-Kudo *et al.* (2005) followed by Chen *et al.* (2011) in 6 studies. While most studies used spiked samples, naturally contaminated samples have been examined. Platforms adopted for these assays are similar to those used in assay development as are the amplicon detection methods (Table 2).

Without enrichment, the reported sensitivity varies greatly, ranging from 2.2 CFU/g to 10^8^ CFU/mL (Table 2). Enrichment (4 h to overnight) has been widely adopted and some studies reported probabilities of detection in lieu of limits of detection. The inclusion of an enrichment step clearly increased the ability of LAMP assays to detect *Salmonella* in food; many reported the successful detection of <1 CFU per test portion (in gram or mL) analyzed (Table 2).

### Application in feed

Six recent studies have described the application of *Salmonella* LAMP assays in animal food matrices (Table 2). Notably, the closed-tube Genie II platform for real-time fluorescence detection of LAMP amplicon uses an extra anneal step, which has been explored recently for duplex detection of two targets by using the distinct annealing temperatures of the LAMP products, as described by Liu *et al.* (2017) for the detection of *Salmonella* and *V. parahaemolyticus* and by D'Agostino *et al.* (2015) for the detection of *Salmonella* and an internal amplification control (IAC). In the latter study, the IAC sequence was designed so that it could be amplified by the same primer set for *Salmonella*, but with increased G:C content, thereby increasing the annealing temperature of the IAC amplicon by 1.6°C. The assay sensitivity, however, was reduced by 1,000-fold with the IAC (D'Agostino *et al.*, 2015). Nonetheless, the ability to incorporate an IAC is especially useful when applying *Salmonella* LAMP assays in animal food, since it takes longer time to reach positive results in animal food compared to human food, suggesting matrix effects are more pronounced in these matrices (Yang *et al.*, 2016). As in human food applications, with enrichment, *Salmonella* LAMP assays could detect a few CFUs per animal food portion analyzed (Table 2).

### Validation studies

Method validation is a critical step before a new method can be adopted for routine use. Despite growing applications of *Salmonella* LAMP assays in food and feed matrices (Table 2), limited effort has been put forth to validate the assay performance against well-established reference methods following international guidelines (AOAC, 2012; ISO, 2016). These validation studies, performed at single laboratory, independent laboratory, and collaborative study (interlaboratory) levels, present rigorous opportunities to test an assay's inclusivity/exclusivity, sensitivity, and probability of detection in a food or feed matrix (AOAC, 2012; ISO, 2016). For instance, in a dog food matrix study, bulk samples are inoculated at low (0.2–2 CFU/25 g) and high (2–10 CFU/25 g) concentrations, mixed well, and aged for at least 2 weeks to best mimic a natural contamination event (AOAC, 2012). The reference method and the alternative method are then applied to detect *Salmonella* using either a paired or unpaired study design (ISO, 2016).

In this context, validations of several commercially available *Salmonella* LAMP detection kits have been completed, including 3M MDA *Salmonella* in raw ground beef and wet dog food (Bird *et al.*, 2013, 2014), 3M MDA 2—*Salmonella* in raw ground beef and creamy peanut butter (Bird *et al.*, 2016), and SAS Molecular Tests *Salmonella* Detection Kit in ground beef, beef trim, ground turkey, chicken carcass rinses, bagged mixed lettuce, and fresh spinach (Bapanpally *et al.*, 2014). Among them, 3M MDA 2—*Salmonella* has been approved for Official Method of Analysis (OMA) by AOAC International (OMA method No. 2016.01).

It is noteworthy that two *Salmonella* LAMP assays geared toward applications in animal food have moved forward with such validation efforts. D'Agostino *et al.* (2016) described the validation of a LAMP/ISO 6579-based method for analyzing soya meal (an animal feed ingredient) for the presence of *Salmonella* spp. through an interlaboratory trial. The alternative method achieved the same percentage correct identification (full agreement) as the reference method, demonstrating its suitability for adoption as a rapid method for identifying *Salmonella* in this matrix. In another study (Domesle *et al.*, 2018), we reported the validation of our *invA*-based *Salmonella* LAMP assay in multiple animal feed and pet food items by closely following the guidelines (AOAC, 2012; FDA, 2015; ISO, 2016). Compared to the reference method, the relative levels of detection for all animal food items fell within the acceptability limits for an unpaired study (Domesle *et al.*, 2018).

## Future Perspectives

In this review, we summarized 100 articles published around the globe between 2005 and 2018 on the development and application of *Salmonella* LAMP assays in various food and feed matrices (Table 2). LAMP has clearly established itself as a powerful alternative to PCR for the rapid, reliable, and robust detection of *Salmonella*, with several assays already successfully validated through multilaboratory studies in specific food and feed matrices.

It is a high possibility that scientific and commercial advancements in the LAMP technology, in general, will propel and shape future developments in this field. This includes the development of new LAMP reagents and new platforms to further capitalize on the two most distinctive characteristics of LAMP, that is, rapidity and simplicity (Mori *et al.*, 2013). Already, we have seen many recent developments in new LAMP reagents, particularly enzymes and master mixes, for example, *Bst* 2.0 and *Bst* 2.0 WarmStart DNA polymerases (New England Biolabs, Ipswich, MA), *Gsp*SSD and *Tin* DNA polymerases and isothermal master mixes (OptiGene Ltd., West Sussex, United Kingdom), and OmniAmp DNA polymerase and LavaLAMP master mixes (Lucigen Corporation, Middleton, WI), which offer better thermostability, higher amplification efficiency, and are thus more amenable to resource-limited and field conditions. Positive results may be obtained within 5 min using some of these reagents. Lyophilized LAMP reagents have been commercialized for some clinical diagnostic kits (Mori *et al.*, 2013), a reagent format that may be adopted by *Salmonella* LAMP detection kits for food and feed in the future.

Multiplex LAMP assays are just beginning to be explored (Mayboroda *et al.*, 2018), using release of quenching technology (Tanner *et al.*, 2012), fluorogenic hybridization (Nyan and Swinson, 2015), endonuclease restriction (Wang *et al.*, 2015), assimilating probes (Kubota and Jenkins, 2015), and annealing temperature differentiation (D'Agostino *et al.*, 2015; Liu *et al.*, 2017) to detect multiple targets in a single reaction tube. The latter two techniques have been applied in *Salmonella* (D'Agostino *et al.*, 2015; Kubota and Jenkins, 2015; Liu *et al.*, 2017). These differ in principle from parallel detection described for many POC microfluidic devices where LAMP reactions for multiple targets are carried out in separate chambers or wells simultaneously. Future developments in chemistries/strategies for multiplex LAMP assays will greatly advance the multiplex LAMP detection of *Salmonella* (multiple genes or pathogens).

Regarding new platform developments, closed-tube, “one-pot” platforms that allow rapid, sensitive, specific, and real-time amplification and detection in small, portable, robust, and user-friendly instruments will be the mainstream. The development and refinement of microfluidic devices (heat control, fluid manipulation, and monitoring method) will continue at a rather fast speed, focusing on full integration of sample preparation, amplification, and detection on one simple, small, user-friendly microdevice. Improvements in sample throughput and field amenability are also desired.

Special considerations should be given when adopting these new advancements in food and feed testing. In terms of assay development, there is currently a paucity of LAMP primers developed for specific *Salmonella* serovars other than *Salmonella* Enteritidis and *Salmonella* Typhimurium. LAMP assays for *Salmonella* serovars that are major animal pathogens are also scarce. Progresses in the areas of viable detection (Lu *et al.*, 2009; Chen *et al.*, 2011; Techathuvanan and D'Souza, 2012) and contamination prevention (Hsieh *et al.*, 2014) have been made and further research is still needed. Simple and effective sample preparation methods, including DNA extraction and storage for field detection are in great demand. Further developments in noninstrumented nucleic acid amplification such as running the assays in a thermos (Kubota *et al.*, 2013) or a pocket warmer (Zhang *et al.*, 2018) will enable field-based food and agricultural diagnostics. Finally, there is an increasing need for matrix-specific validation of newly developed methods. Such validations should follow international guidelines before the methods can be adopted for routine use in food and feed testing.

**Disclaimer:** The views expressed in this article are those of the authors and do not necessarily reflect the official policy of the Department of Health and Human Services, the U.S. Food and Drug Administration, or the U.S. Government. Reference to any commercial materials, equipment, or process does not in any way constitute approval, endorsement, or recommendation by the Food and Drug Administration.
